# Clinical Efficacy of a Novel Therapeutic Principle, Anakoinosis

**DOI:** 10.3389/fphar.2018.01357

**Published:** 2018-11-28

**Authors:** Daniel Heudobler, Michael Rechenmacher, Florian Lüke, Martin Vogelhuber, Sebastian Klobuch, Simone Thomas, Tobias Pukrop, Christina Hackl, Wolfgang Herr, Lina Ghibelli, Christopher Gerner, Albrecht Reichle

**Affiliations:** ^1^Department of Internal Medicine III, Hematology and Oncology, University Hospital Regensburg, Regensburg, Germany; ^2^Department of Surgery, University Hospital Regensburg, Regensburg, Germany; ^3^Department Biology, Universita' di Roma Tor Vergata, Rome, Italy; ^4^Faculty Chemistry, Institut for Analytical Chemistry, University Vienna, Vienna, Austria

**Keywords:** Anakoinosis, communicative reprogramming, transcriptional modulators, metronomic low-dose chemotherapy, glitazones, all-trans retinoic acid, COX-2 inhibitor, master modulators

## Abstract

Classic tumor therapy, consisting of cytotoxic agents and/or targeted therapy, has not overcome therapeutic limitations like poor risk genetic parameters, genetic heterogeneity at different metastatic sites or the problem of undruggable targets. Here we summarize data and trials principally following a completely different treatment concept tackling systems biologic processes: the principle of communicative reprogramming of tumor tissues, i.e., **anakoinosis** *(ancient greek for communication)*, aims at establishing novel communicative behavior of tumor tissue, the hosting organ and organism via re-modeling gene expression, thus recovering differentiation, and apoptosis competence leading to cancer control – in contrast to an immediate, “poisoning” with maximal tolerable doses of targeted or cytotoxic therapies. Therefore, we introduce the term “Master modulators” for drugs or drug combinations promoting evolutionary processes or regulating homeostatic pathways. These “master modulators” comprise a broad diversity of drugs, characterized by the capacity for reprogramming tumor tissues, i.e., transcriptional modulators, metronomic low-dose chemotherapy, epigenetically modifying agents, protein binding pro-anakoinotic drugs, such as COX-2 inhibitors, IMiDs etc., or for example differentiation inducing therapies. Data on 97 anakoinosis inducing schedules indicate a favorable toxicity profile: The combined administration of master modulators, frequently (with poor or no monoactivity) may even induce continuous complete remission in refractory metastatic neoplasia, irrespectively of the tumor type. That means recessive components of the tumor, successively developing during tumor ontogenesis, are accessible by regulatory active drug combinations in a therapeutically meaningful way. Drug selection is now dependent on situative systems characteristics, to less extent histology dependent. To sum up, anakoinosis represents a new substantive therapy principle besides novel targeted therapies.

## Introduction

Each cancer treatment strategy is based on simplifying methodological assumptions (Reichle, 2010). One basic consideration relies on the suggestion that essential tumor-promoting pathways are ubiquitously distributed at metastatic tumor sites and should be shut off, or single cell types within a tumor disease should be knocked out via specific targets. Molecular tumor boards and corresponding “intelligent” treatment algorithms are the logic consequence of this assumption.

Therapeutic limitations of classic targeted tumor therapies are (molecular-) genetic heterogeneity at metastatic sites, poor risk genetic parameters as well as context-dependent validity and denotation of tumor-promoting aberrations and targets (dependent of the genetic background) (Box 1). This fact may be exemplified by context-dependent efficacy of B-RAF inhibitors in different tumor histologies (Flaherty et al., 2010; Kopetz et al., 2015).

Box 1 **Explanation of communication terms.**

**Table:** 

**Communication-associated terms**	**Explanation**
Anakoinosis	Communicatively reprogramming biologic systems, here tumor systems. **Anakoinosis** aims at establishing novel communicative behavior of tumor tissue
Validity and denotation	Validity of systems objects, functions and hubs: Availability on demand at distinct systems stages; denotation: Current functional impact at a distinct systems stage, e.g., of potentially tumor-promoting pathways. In the bio-world, presence and functioning of an object (e.g., an enzyme), respectively
Rationalizations	Describe the physical organization of tumor-associated normative notions (e.g., hallmarks of cancer); are to some degree histology- and genotype-independent; may be re-directed and reorganized by anakoinosis
Metabolism of evolution	The sum of **extrinsically**, i.e., therapeutically, and **intrinsically** inducible **evolutionary processes** within the tumor environment (tumor stroma, hosting organ, distant organ sites)
Modularity	Modularity describes the degree and specificity to which systems' objects, i.e., cells, pathways, molecules, therapeutic targets etc. may be communicatively rededicated by anakoinosis
Pro-anakoinotic therapeutic tools (examples)	Transcriptional modulators Nuclear receptor agonists, antagonists Metronomic low-dose chemotherapy Cyclooxygenase-2 inhibitors IMiDs Arsenic trioxide Liposomal encapsulated small oligonucleotide encoding small activating RNAs

Here we summarize trials principally following a completely different treatment concept. All the mentioned schedules are tackling systems biologic processes, such as dysregulated homeostatic pathways in tumors or are recalling or perfectioning patterns of evolutionary processes (“metabolism of evolution”) provided by single cell types and cell systems in a tumor (Box 1). Thus, also drugs, particularly drug combinations, may be introduced with biomodulatory activity, so called master modulators of tissues, promoting evolutionary processes or regulating homeostatic pathways for treating metastatic and refractory metastatic disease or hematologic neoplasia (Hart et al., 2015).

**Master modulators of tumor tissues**, such as transcriptional modulators, hormones, cytokines, vitamins, epigenetically active drugs, metronomic low-dose chemotherapy and protein-binding drugs cyclooxygenase-2 (COX-2) inhibitors, IMiDs, arsenic trioxide etc. are aiming at reconditioning tumor tissue into a controlled phenotype, thereby diversifying palliative care, or even inducing continuous complete remission (Table 1; Box 1; Hart et al., 2015). Master modulators may therapeutically cope with different, but iterative patterns and physical constitutions of hallmarks of cancer supported by quite heterogeneous tumor genotypes. Those different patterns of acquired chromosomal aberrations may support a unique hallmark, exemplified in acute leukemias by the rapidly displacing growth in the bone marrow.

**Table 1:** Master modulators including transcriptional modulators in 97 clinical trials: Master modulators are transcriptional modulators (hormones, cytokines, vitamins etc.), metronomic low-dose chemotherapy, protein-binding drugs (arsenic trioxide, COX-2 inhibitors, IMiDs etc.), metabolic active drugs, such as PPAR gamma/α agonists, statins, and metformin (interventional statin and metformin trials are not included in the review; also, not included nuclear receptor antagonists).

**Ninety-seven studies including master modulators (25 histologic entities). Dysregulated transcription programs: Cancer cells are highly dependent on regulators of gene expression**.					
**Schedule of master modulators**		**No of studies**	**Drugs (approved in bold)**		**Comments**
**Transcriptional modulator** ***as monotherapy*** (Light blue Table I-VIII)	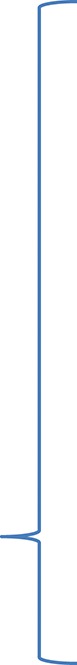	**26**	• **Vitamin D** • **Interferon-alpha** • **LHRH agonist** • **Somatostatin** • **All-trans retinoic acid**	•**Estrogen** •*Six trials on* **pioglitazone**, rosiglitazone, troglitazone • **Bexaroten** •(MTL-CEBPA; IDH inhibitor)	**No monoactivity:** •**Glitazones in four** • **histologic tumor types** • Estrogen
**Simultaneous administration of two or more transcriptional modulators plus/minus additional master modulators** (without metronomic chmotherapy) (Green Table II-V)		**17**			
Agonistic acting drugs			•**Dexamethasone/**combined with **IMiD** •**LHRH agonist/**combined with **Vitamin D** or **dexamethasone/somatostatin** or **Interleukin-2 (IL-2)** •**IFN-alpha/** combined with **somatostatin** or **dexamethasone** or **IL-2** • **Somatostatin**/ combined with estrogen or **IFN-alpha** •**All-trans retinoic acid/** combined with **arsenic trioxide** or **interferon-alpha**		Combinations may be equally efficacious compared to standard chemotherapy ***(Renal clear cell carcinoma, castration-resistant prostate cancer, neuroendocrine tumors, acute promyelocytic leukemia: phase III trial)***
**One transcriptional modulator** plus/minus IMiD, COX-2 inhibitor ***plus metronomic low-dose chemotherapy*** (Violet Table I-VIII)		**13**	•Troglitazone or **pioglitazone** • **Interferon-alpha** • **Thalidomide/**combined with **celecoxib** •**Pioglitazone/**combined with **COX-2 inhibitor**		Thiazolidinediones highly efficacious in respective combinations
**Multiple transcriptional modulators** plus/minus other master modulators ***plus metronomic low-dose chemotherapy*** (Red Table I-III)		**5**	•**Pioglitazone**/ combined with **interferon-alpha** or **dexamethasone** •**Vitamin D/fenofibrate/retinoic acid** *(COMBAT trial)*		Continuous complete remission, active chronification in refractory disease possible
**Master modulators** *plus targeted therapy* (Brown Table I-VIII)		**18**	**Propranolol; temsirolimus; everolimus; imatinib; bortezomib**; bcl2- antisense; **blinatumumab; sunitinib; bevacizumab**; **tamoxifen; letrozol**; denileukin difitox; veliparib		**Randomized phase III trial, imatinb/pioglitazone**
**Master modulator(s)** *plus pulsed chemotherapy* (Dark blue Table II-VIII)		**14**	•**Prednisolone** •**Methylprednisolone/IL-2** •**Somatostatin** •**Retinoic acid**	•Melatonin •**Vitamin D** •**LHRH agonist**	Less efficacious combinations •Somatostatin/retinoic acid/melatonin/VitD/bromocriptin; •Retinoic acid/interferon-alpha •Metronomic low-dose chemotherapy
**Transcriptional modulator(s)** ***plus demethylating or deacetylating agent*** (Yellow Table VI)		**4**	• **Azacitidine/all-trans retinoic acid/pioglitazone** • **Valprionic acid/ retinoic acid or bexaroten** • **Vorinostat/bexaroten/fenofibrate**		Induction of complete remission possible in refractory disease

Basis for the concerted regulatory activity profile of master modulators are during tumor ontogenesis developing dysregulated transcription programs, networks of pathways and interlaced communication routes among cancer cells, adjacent stroma cells, tumor bearing organ and organism.

Communicative reprogramming of tumor tissues, i.e., **anakoinosis**, aims at establishing novel communicative behavior of tumor tissue, the hosting organ and organism via re-modeling gene expression, thus recovering differentiation, and apoptosis competence leading to cancer control (Box 1; Hart et al., 2015).

The presented tool of clinical observations on anakoinosis inducing therapy approaches reveals that tumor tissue provides an extensive design space, including the interaction of tumor and tumor bearing organ and organism (Hart et al., 2015). The biological necessity of tumor site to respond with clinically relevant changes in tumor behavior following exposure to master modulators that means anakoinosis-inducing drugs, is predefined by not necessarily histologically determined prerequisites guiding communication.

## Master modulators, the backbone of anakoinosis inducing therapies

### Broad repertoire of possible approaches for inducing anakoinosis: diversity of master modulators of tumor tissues

The instruments for inducing anakoinosis are multifaceted and still insufficiently explored. An important distinguishing characteristic, in contrast to classic targeted therapy, is the generally observed minor monoactivity, but frequently “concerted” activity profile of single pro-anakoinotic drugs (Tables I–VIII), the possibility for successfully administering agonistic, immunomodulatory and anti-inflammatory drugs and the modest toxicity profile.

**Table IA:** Communicative reprogramming of tumor disease.

	***Glitazones IA***					
**Neoplasia**	**No pts**	Chemotherapy (metronomic)*	**Transcriptional modulators**	**Small molecule**	**Best response**	**Publication**
**SARCOMAS**						
Liposarcomas, intermediate to high-grade (case reports)	–	–	• **Troglitazone**	–	Histological and biochemical differentiation	Tontonoz et al., 1997
Liposarcoma	3	Trofosfamide*	• **Troglitazone**	**–**	Lineage-appropriate differentiation can be induced pharmacologically in a human solid tumor.	Demetri et al., 1999
Liposarcoma (Phase II study)	12	**–**	• **Rosiglitazone**	–	Rosiglitazone is not effective as an antitumoral drug in the treatment of liposarcomas	Debrock et al., 2003
Kaposi sarcoma, refractory	1	Trofosfamide*	• **Pioglitazone**	• **COX-2 inhibitor**	**Partial remission**	Coras et al., 2004
(Hem-)angiosarcomas	12)	Trofosfamide*	• **Pioglitazone**	• **COX-2 inhibitor**	**Continuos complete remission**	Vogt et al., 2003
Angiosarcoma	7	Vinblastine* Methotrexate*	**–**	• **Propranolol**	**Complete remission**	Pasquier et al., 2016
**BREAST CANCER**						
Refractory breast cancer (Phase II study)	22	–	• **Troglitazone**	–	No significant effect	Burstein et al., 2003
**MELANOMA**						
Melanoma III (versus DTIC), phase II ClinicalTrials.gov:NCT01614301	**6**	Trofosfamide*	• **Pioglitazone**	**Temsirolimus** **COX-2 inhibitor**	**Partial remission, Resolution of cachexia**	Hart et al., 2016
**MELANOMA (RANDOMIZED)**						
Melanoma II Arm M Arm A/M	35 32	Trofosfamide*Trofosfamide*	**–** **Pioglitazone**	-• **COX-2 inhibitor**	Stable disease **Partial remission**	Reichle et al., 2007b
**HEPATOCELLULAR CARCINOMA**						
Hepatocellular carcinoma	38	• Capecitabine*	• **Pioglitazone**	• **COX-2 inhibitor**	**Partial remission**	Walter et al., 2017
**CHOLANGIOCELLULAR CARCINOMA**						
Cholangiocellular carcinoma	21	Trofosfamide*	• **Pioglitazone**	• **COX-2 inhibitor**	**Partial remission**	Reichle et al., 2010
**COLORECTAL CANCER**						
Chemotherapy-resistant metastatic colorectal cancer (phase II study)	25	**–**	• **Troglitazone**	**–**	Not active for the treatment of metastatic colorectal cancer	Kulke et al., 2002
**RENAL CLEAR CELL CARCINOMA** (**HISTORIC COMPARISON**)						
Renal clear cell carcinoma, relapsed	18	Capecitabine*	• **Pioglitazone**	• **COX-2 inhibitor**	**Partial remission**	Reichle et al., 2007a
Renal clear cell carinoma, relapsed	33	Capecitabine*	**Pioglitazone** **Interferonalpha**	• **COX-2 inhibitor**	**Continuous complete remission**	Walter et al., 2012; Hart et al., 2016

*Anakoinotic therapy approaches sorted by transcriptional modulator and tumor disease. For comparison of clinical results on pro-anakoinotic therapies, the tables additionally indicate data on metronomic chemotherapy alone. **Light blue**: Monotherapy with transcriptional modulators; **green:** Several transcriptional modulators or master modulators (without metronomic chemotherapy); **violet:** Metronomic chemotherapy plus transcriptional modulator/ or other master modulators; **red:** Metronomic chemotherapy and multiple transcriptional modulators or master modulators; **brown:** Master modulators plus targeted therapy; **dark blue:** Pulsed chemotherapy plus master modulator(s); **yellow:** Demethylating agent/deacetylating agent plus transcriptional modulators*.

**Table IB:** Communicative reprogramming of tumor disease.

	***Glitazones IB***					
**Neoplasia**	**No pts**	Chemotherapy (metronomic)*	**Transcriptional modulators**	**Small molecule**	**Best response**	**Publication**
**PROSTATE CANCER**						
Prostate cancer	41	–	• **Troglitazone**	–	Lengthened stabilization of prostate-specific antigen	Mueller et al., 2000
Castration-resistant prostate cancer	61	• Treosulfan*	• **Pioglitazone**, •**Dexamethasone**	• **COX-2 inhibitor** • **Imatinib**	**Long-term tumor control at minimal disease**	Vogelhuber et al., 2015
Castration-resistant prostate cancer	36	• Capecitabine*	• **Pioglitazone,** •**Dexamethasone**	• **COX-2 inhibitor**	**Long-term tumor control**	Vogt T. et al., 2006; Walter B. et al., 2010
**PROSTATE CARCINOMA (RANDOMIZED)**						
Rising serum prostate-specific antigen level after radical prostatectomy and/or radiation therapy	106	-	• **Rosiglitazone** ***versus*** • **Placebo**	-	Rosiglitazone did not increase PSA doubling time or prolong the time to disease progression	Smith et al., 2004
**GASTRIC CANCER (RANDOMIZED)**						
Gastric cancer Arm A/MArm M	21 21	• Capecitabine* • Capecitabine*	• **Pioglitazone** -	• **COX-2 inhibitor** -	**Partial remission, pioglitazone no impact**	Reichle et al., 2009
**MULTIPLE MYELOMA**						
Multiple myeloma, third-line Clinicaltrials.gov, NCT001010243	6	• Treosulfan*	• **Pioglitazone,** • **Dexamethasone**	• **Lenalidomide**	**Complete remission**	Reichle et al., 2012
**LANGERHANS CELL HISTIOCYTOSIS**						
Langerhans cell histiocytosis, refractory	2+7	• Trofosfamide*	• **Pioglitazone** • **Dexamethasone**	• **COX-2 inhibitor**	**Continuous complete remission**	Reichle et al., 2005; Hart et al., 2015; Heudobler et al., 2016
**HODGKIN LYMPHOMA**						
Hodgkin lymphoma, refractory	3	• Treosulfan*	• **Pioglitazone**, •**Dexamethasone**	• **COX-2 inhibitor** • **Everolimus**	**Continuous complete remission**	Ugocsai et al., 2016
**CHRONIC MYELOCYTIC LEUKEMIA**						
Chronic myelocytic leukemia without moleclar CR	24	–	• **Pioglitazone**	• **Imatinib**	**Molecular complete remission (54%)**	Prost et al., 2015
**GLIOBLASTOMA**						
Glioblastoma, refractory	*14*	• Capecitabine*	• **Pioglitazone**	• **COX-2 inhibitor**	Disease stabilization	Hau et al., 2007
**GLIOBLASTOMA (RANDOMIZED)**						
Glioblastoma	85	Temozolomide*(Tem)*versus*Dose dense Tem	• **Sequentially 13-*****cis***-retinoic acid*	**-**	No benefit of metronomic chemotherapy in maintenance	Clarke et al., 2009

**Table II:** Communicative reprogramming of tumor disease.

	**Glucocorticoids**					
**Neoplasia**	**No pts**	**Chemotherapy**	**Transcriptional modulators**	**Small molecule**	**Best response**	**Publication**
**MULTIPLE MYELOMA (RANDOMIZED)**						
Multiple myeloma	–	–	• **Dexamethasone** • **versus Combination**	•+ **different targeted therapies**	Most combinations are superior to dexamethasone alone	van Beurden-Tan et al., 2017
Relapsed multiple myeloma	353	–	• **Dexamethasone** • **versus Dexamethasone only**	• **Lenalidomide**	Lenalidomide plus dexamethasone is superior	Weber et al., 2007
Relapsed or refractory multiple myeloma	351	–	• **Dexamethasone Versus** • **Dexamethasone only**	• **Lenalidomide**	Lenalidomide plus dexamethasone is more effective than high-dose dexamethasone alone	Dimopoulos et al., 2007
Relapsed and refractory multiple myeloma	302	–	• **Dexamethasone** • **versus** **high-dose dexamethasone**	• **Pomalidomide** • **No pomalidomide**	Pomalidomide plus low-dose dexamethasone, new treatment option	San Miguel et al., 2013
Relapsed multiple myeloma	669	–	• **Bortezomib** **or high-dose dexamethasone**	• **Bortezomib**	Bortezomib is superior to high-dose dexamethasone	Richardson et al., 2005
Advanced multiple myeloma	224	–	• **Dexamethasone** ± **Oblimersen sodium**	• **Oblimersen sodium (bcl-2 antisense oligonucleotide**	No significant differences between the two groups in TTP or objective response rate	Chanan-Khan et al., 2009
**LYMPHOMA (RANDOMIZED)**						
Elderly patients with aggressive non-Hodgkin's lymphoma	453	Chemotherapy AChemotherapy B	• **Prednisolone** • **Prednisolone**	–	Slightly longer survival was observed for patients treated with an **anthracycline-containing regimen**	Bastion et al., 1997
**LYMPHOMA**						
Refractory chronic lymphocytic leukemia	14	-	• **High dose methyl- prednisolone**	**–**	HDMP may be beneficial in the treatment of refractory CLL but is of no value in CLL/PL.	Thornton et al., 1999.
Diffuse large B-cell lymphoma	21	–	• **Dexamethasone (supportive)**	• **Blinatumomab**	Complete remission due to blinatumumab	Viardot et al., 2016
Hodgkin disease	Review	Chemotherapy	• **Prednisolone**	±	Continuous complete remission, decisive is kind of chemotherapy	Ansell, 2015
**ACUTE LYMPHOBLASTIC LEUKEMIA (RANDOMIZED)**						
Childhood acute lymphoblastic leukemia	1603	–	• **Dexamethasone versus** • **Prednisolone**	–	**Dexamethasone led to a significant decrease in the risk of relapse for all risk-groups**	Mitchell et al., 2005

**Table III:** Communicative reprogramming of tumor disease.

	**Vitamin D**					
**Neoplasia**	**No pts**	Chemotherapy (metronomic*)	**Transcriptional modulators**	**Small molecule**	**Best response**	**Publication**
**CANCER**						
Reducing cancer risk, progression	–	–	• **Vitamin D**	**–**	Possible therapeutic benefit	Feldman et al., 2014
Relapsing/refractory malignancies (COMBAT:“Combined oral metronomic biodifferentiating anti-angiogenic treatment“)	74	Temozolomide* Etoposide*	• **Vitamin D** • **Fenofibrate** • **Retinoic acid**	• **Celecoxib**	**Complete remission**	Zapletalova et al., 2012
Cancer	Review	–	• **LHRH agonist**, • **Vitamin D**	**–**	Osteoporosis prophylaxis	Nicolini et al., 2016
**SARCOMA**						
Kaposi sarcoma	8	**–**	• **Vitamin D(3) receptor agonist**	–	The antitumor activity: topical application	Masood et al., 2000
	***S*****OMATOSTATIN analog, melatonin**					
**Neoplasia**	**No**	**Chemo-therapy**	**Transcriptional modulators**	**Small molecule**	**Best response**	**Publication**
**NEUROENDOCRINE TUMORS**						
Neuroendocrine tumors	Meta-analysis	**–**	**Somatostatin analog**	-	Stable disease: 67% of patients	Sidéris et al., 2012
Neuroendocrine tumors	Meta-analysis		**Somatostatin analog**	**mTOR inhibitor**	**Approved**	Bousquet et al., 2012
Metastatic endocrine tumors	Review	–	**Low-dose subcutaneous interleukin-2, melatonin**	**–**	**Partial response**	Lissoni et al., 1995
Gastroenteropancreatic neuroendocrine carcinoma **(randomized)**	Review	–	• **Interferon-alpha** • **Somatostatin analog**	–	No statistically significant survival benefit compared to single agent	Fazio et al., 2007
Neuroendocrine tumors **(randomized)**	80	**–**	• **Lanreotide versus** • **Interferon-alpha versus** • **Lanreotide, IFN-alpha**	–	No difference in response	Faiss et al., 2003
**CASTRATION-RESISTANT PROSTATE CANCER (RANDOMIZED)**						
Castration-resistant prostate cancer **(randomized)**	40	**Estramustine, etoposide** ***versus***	• **LHRH analog** • **Somatostatin analog** • **Dexamethasone**	–	**Equally effective compared to salvage chemotherapy**	Dimopoulos et al., 2004

**Table IV:** Communicative reprogramming of tumor disease.

	**Interferon-alpha**					
**Neoplasia**	**No pts**	Chemotherapy (metronomic)*	**Transcriptional modulators**	**Small molecule**	**Best response**	**Publication**
**METASTATIC RENAL CELL CARCINOMA**						
Renal clear cell carinomaPhase I trial	12	–	• **Interferon alpha2b** • **Liposome-encapsulated all-trans retinoic acid**	–	**Partial response**	Goldberg et al., 2002
**RENAL CELL CARCINOMA RANDOMIZED**						
Renal clear cell carinoma	750	–	• **Interferon-alpha** ***versus***	• **Sunitinib**	Progression-free survival superior compared to IFNalpha	Motzer et al., 2007
Metastatic renal carcinoma(randomized)	350	–	• **Interferon-alpha** ***versus*** • **Methyl-progesterone acetat**	–	Improvement in median survival of 2.5 months (MPA 6 months, interferon-alpha 8.5 months)	1999
Metastatic renal cell carcinoma (randomized)	649	–	• **Interferon-alpha** ***versus*** • **Interferon-alpha plus**	• **Bevacizumab**	**Interferon-alpha: Significant improvement in progression-free survival (approved)**	Escudier et al., 2007
Metastatic renal cell carcinoma	192	–	• **High-dose (HD) IL-2** ***versus*** • **Il-2 plus IFN-alpha**	**–**	**HD IL-2 should remain the preferred therapy for selected patients with metastatic renal cell carcinoma (app**roved)	McDermott et al., 2005
**MELANOMA**						
Melanoma, a systematic review	–	–	•**Interferon-alpha** (maintenance)	–	No convincing evidence of a survival benefit	Di Trolio et al., 2015
Melanoma, resected stage III(randomized)	1256	–	• **Interferon-alpha, adjuvant** ***versus*** **observation**	-	Adjuvant PEG-IFN-α-2b for stage III melanoma: positive impact on RFS (marginally significant)	Eggermont et al., 2008
**FOLLICULAR LYMPHOMA (RANDOMIZED)**						
**Follicular lymphoma**(randomized)	77	Chemotherapy ± rituximab	•±**interferon-alpha maintenance**	**–**	Improved PFS and EFS without an impact on OS	Herold et al., 2015
Refractory/relapsed cutaneous T-cell lymphoma(randomized)	370	Low-dose MTX*–	• **Interferon-alpha (IFN-a)** ***versus*** • **IFN-a plus Retinoids**	–	**Overall survival identical**	Aviles et al., 2015
**MULTIPLE MYELOMA**						
Multiple myeloma	402	–	• **Interferon-alpha** (maintenance after melphalan, prednisone)	–	**Interferon improves progression-free and overall survival who respond to melphalan and prednisone**	Browman et al., 1995
**CHRONIC MYELOCYTIC LEUKEMIA**						
Chronic myelocytic leukemia	–	–	• **Interferon-alpha pegylated**	• **Imatinib**	**Increases molecular response rates**	Simonsson et al., 2011
**EPITHELIAL NEOPLASIA**						
**Corneal epithelial neoplasia**	89	–	• **Retinoic acid and topical interferon alfa-2b**	**–**	**Complete remission**	Krilis et al., 2012

**Table V:** Communicative reprogramming of tumor disease.

	**Estrogene, Luteinizing hormone-releasing hormone agonist**					
**Neoplasia**	**No pts**	**Chemo-therapy**	**Transcriptional modulators**	**Small molecule**	**Best response**	**Publication**
**PROSTATE CANCER**						
Androgen independent prostate cancer (Phase II study)	45	-	• **High dose conjugated estrogen** (Premarin)	**-**	Prostate specific antigen decreases of 50% or greater in 25% of patients with androgen independent prostate cancer	Pomerantz et al., 2007
Castration-refractory prostate cancer	38	-	• **LHRH agonist**, • **Dexamethasone** • **Somatostatin analog**	**-**	**Durable objective responses**	Koutsilieris et al., 2006
Castration-resistant prostate cancer	Review	-	• **Somatostatin analogs** • **Estrogens**	**-**	**Median survival of 10 months**	Sciarra et al., 2004
**ENDOMETRIAL CANCER**						
Endometrial cancer	16 608	-	• **Continuous combined estrogen plus progestin**	**-**	Statistically non significant reduction in deaths from endometrial cancer in the estrogen plus progestin group	Chlebowski et al., 2016
**BREAST CANCER**						
Breast cancer, metastatic	32	-	• **High-dose estrogen**	**-**	Antitumor effects in breast cancer patients heavily pretreated with endocrine therapy	Lønning et al., 2001
**BREAST CANCER (RANDOMIZED)**						
Adjuvant endocrine therapy in premeno-pausal breast cancer	927	-	• **Luteinizing hormone-releasing hormone agonist**	• Tamoxifen	The combination of goserelin and tamoxifen is not superior to either modality alone	Sverrisdottir et al., 2011
**BREAST CANCER**						
Breast cancer, prostate cancer	-	-	• **LHRH agonist**	**-**	**Long-term tumor control**	Sharma et al., 2008
Estrogen receptor (ER)-alpha positive metastatic breast cancer	Review	-	• **LHRH analog** • **Interleukin-2**	**-**	Randomized trials are necessary	Nicolini et al., 2016
	**Differentiation inducing small molecules (exemplarily)**					
**Neoplasia**	**No pts**	**Chemo-therapy**	**Transcriptional modulators**	**Small molecule**	**Best response**	**Publication**
**Hepatocellular carcinoma**	19	**-**	**-**	MTL-CEBPA, liposomal saRNA	**Partial remission**	Sarker et al., 2018
**Acute myelocytic leukemia, IDH mutated**	179	**-**	**-**	Isocitrat-Dehydro-genase, IDH inhibitor	**Durable remissions**	DiNardo et al., 2018

**Table VI:** Communicative reprogramming of tumor disease.

	**Retinoic acid**					
**Neoplasia**	**No pts**	**Epigenetic therapy**	**Transcriptional modulators**	**Small molecule**	**Best response**	**Publication**
**CANCER**						
Cancer therapy	**-**	-	• **All-trans retinoic acid (RA)**	-	Disruption of RA signaling pathways: Hematological and non-hematological malignancies	Altucci et al., 2007; Di Masi et al., 2015
Advanced cancer	28	-	• **9-cis retinoic acid**	-	Recommended 140 mg/m2 once-daily	Miller et al., 1996
**ACUTE MYELOID LEUKEMIA**						
Acute promyelocytic leukemia	263	Chemotherapy plus all-trans retinoic acid vs.	• **All-trans retinoic acid (ATRA)** • **Arsenic trioxide (ATO)**	-	**Continuous complete remission with ATRA, ATO Approved therapy**	Efficace et al., 2014
Refractory and high-risk acute myeloid leukemia (AML)	**–**	**Valproic acid (VPA)**	• **All-trans retinoic acid**	–	In conclusion, VPA-ATRA treatment is well tolerated and induces phenotypic changes of AML blasts through chromatin remodeling	Cimino et al., 2006
Acute myelocytic leukemia, refractory	5	**Azacytidine (low-dose**)	• **Pioglitazone**, • **All-trans retinoic acid**	–	**Complete remission**	Thomas et al., 2015; Heudobler et al., 2018a
**LYMPHOMA (RANDOMIZED)**						
T-cell lymphoma	**377**	-Low-dose MTX*	• **IFN-alpha plus retinoid** ***versus*** **Interferon-alpha**	**–**	**Overall complete response rate: 80% in both arms**	Aviles et al., 2015
	**Bexarotene (retinoic X receptor agonist)**					
**CUTANEOUS T-CELL LYMPHOMA**						
Cutaneous T-cell lymphoma, phase I	23	**Vorinostat**	• **Bexarotene**	–	Feasible if lower doses of each drug are administered relative to the product label monotherapy doses	Dummer et al., 2012
Refractory cutaneous T-cell lymphoma	–	–	• **Bexarotene**	**–**	**Complete remission; approved**	Querfeld et al., 2006
Mycosis fungoides/Sézary syndrome	–	–	• **All-trans retinoic acid** •***versus*** **bexarotene**	**–**	Equally efficacious (historic comparison)	Querfeld et al., 2004
Cutaneous T-cell lymphoma, phase I	14	–	• **Bexarotene**	**Denileukin diftitox (IL-2)**	**Complete remission**	Foss et al., 2005
Tumor-stage mycosis fungoides	1	**Vorinostat**	• **Bexarotene** • **High-dose fenofibrate**	**–**	**Complete remission**	Steinhoff et al., 2008
**CANCER**						
Cancer	52	–	• **Retinoid X receptor ligand**	**–**	**Partial remission**	Miller et al., 1997
**NON-M3 ACUTE MYELOID LEUKEMIA**						
Acute myeloid leukemia	27	–	• **Bexarotene**, (phase I)	–	Evidence of antileukemic activity	Tsai et al., 2008

**Table VII:** Communicative reprogramming of tumor disease.

	**Transcriptional modulators plus pulsed chemotherapy**					
**Neoplasia**	**No pts**	**Chemotherapy**	**Transcriptional modulators**	**Small molecule**	**Best response**	**Publication**
**LUNG CANCER**						
Metastatic lung cancer(phase I)	16	Cisplatin and epidoxorubicin ***plus***	• **Medroxypro-gesterone acetate** • **Recombinant interleukin-2**	**-**	No significant relieve of cancer-related cachexia symptoms. 64% objective response	Mantovani et al., 2000
Adenocarcinoma, lung, heavily pretreated	23	Cyclophosphamide ***plus***	• **Somatostatin**, • **Retinoids**, • **Melatonin**, • **Vitamin D**, • **Bromocriptine**	**-**	Improved disease-related symptoms	Norsa and Martino, 2007
**CANCER (RANDOMIZED)**						
Solid tumors (meta-analysis of randomized controlled trials)	-	Concurrent chemo-therapy or radio-therapy	• **Melatonin**	-	Melatonin as adjuvant therapy: Substantial im-provements in tumor remission, 1-year survival, alleviation of radiochemo-therapy-related effects	Wang et al., 2012
**LYMPHOMA (RANDOMIZED)**						
Lymphoma: A Prospective Evaluation in SWOG and LYSA Studies.	777	Chemo-immune therapy	• **Low versus normal vitamin D levels in serum**	**-**	**Low serum vitamin D levels are associated with inferior survival in follicular lymphoma**	Kelly et al., 2015
**PROSTATE CANCER (RANDOMIZED)**						
Androgen-independentprostate cancer	70	Docetaxel	• **Doxercalciferol**	-	Daily doxercalciferol with weekly docetaxel did not enhance PSA response rate or survival	Attia et al., 2008
**COLO-RECTAL CANCER** (**RANDOMIZED**)						
Advanced colorectal cancer	3254	5-Fluorouracil	• **Interferon-alpha**	-	Alpha-IFN does not increase the efficacy of 5FU or of 5FU + LV	Hill et al., 1995; Thirion et al., 2001
**CERVICAL CARCINOMA (RANDOMIZED)**						
Cervical carcinoma(randomized)	209	Cisplatin *plus*	• **Retinoic acid** +**/- Interferon-alpha**	**-**	No survival benefit for the combination	Basu et al., 2016
**CERVICAL SQUAMOUS CELL CARCINOMA**						
Metastatic cervical squamous cell carcinoma: Phase II trials	33	Cisplatin *plus*	• **Interferon-alpha** • **Retinoids**	-	**Objective response**	Braud et al., 2002
**SUPPORTIV**						
**Fertility preservation in women with breast cancer**	-	Polychemotherapy	• **LHRH agonist**	**-**	Fertility preservation, tumor therapy	Taylan and Oktay, 2017

**Table VIII:** Communicative reprogramming of tumor disease.

	**Metronomic chemotherapy (selected randomized trials)**					
**Neoplasia**	**No pts**	Metronomic* chemotherapy	**Transcriptional modulators**	**Small molecule**	**Best response**	**Publication**
**Glioblastoma (RANDOMIZED)**						
**Glioblastoma (randomized)**	85	Temozolomide*vs. Dose dense temozolomide	• **Sequentially 13-*****cis***-retinoic acid*	**-**	No benefit of metronomic chemotherapy in maintenance	Clarke et al., 2009
**OSTEOSARCOMA (RANDOMIZED)**						
Osteosarcoma(adjuvant) **(randomized trial)**	132 157	• Pulsed chemotherapy plus metronomic chemotherapy*versus • Pulsed chemotherapy	**–**	-	No difference in event-free survival	Senerchia et al., 2017
**BREAST CANCER (RANDOMIZED)**						
**Elderly breast cancer patients (randomized)**	114	± Cyclophosphamide*	**–**	• **Letrozol**	Advantage for combination in ductal carcinomas (first-line)	Bottini et al., 2006
**Triple-negative breast cancer (randomized)**	45	Cyclophosphamide*	**–**	± **Veliparib**	No benefit	Kummar et al., 2016
**Her2 negative breast cancer (randomized)**	147	Capecitabine* Cyclophosphamide*vs. Pulsed paclitaxel	**–**	+ **Bevacizumab in each arm**	Response rate 50 ***vs***. 58%, (Not significant)	Rochlitz et al., 2016
**COLO-RECTAL CANCER (RANDOMIZED)**						
**Colo-rectal cancer (randomized)**	558	• Capecitabine* • Observation	**–**	• **Bevacizumab** • Observation	**PFS improved for maintenance therapy from 8.5 to 11.7 months**	Simkens et al., 2015
**Palliative therapy pediatric cancer (randomized** ***vs***. **placebo)**	108	• Etoposide* plus Cyclophosphamide* • Placebo	**–**	• **Celecoxib plus thalidomide** • Placebo	PFS and OS not significant different	Pramanik et al., 2017

Starting point for the current review are a series of systematically developed clinical trials on refractory metastatic tumor diseases including at least one nuclear receptor agonist and metronomic low-dose chemotherapy or epigenetically active drugs as pro-anakoinotic therapy approaches (Hart et al., 2015, 2016; Walter et al., 2017).

Pro-anakoinotic schedules include epigenetically and transcriptionally active drugs, such as agonists of nuclear transcription factors (glitazones, all-trans retinoic acid, bexarotene, glucocorticoids, vitamin D etc.), but also cytokines (e.g., interferon-α, Interleukin-2 etc.) and vitamins. Transcriptional modulators have in common that they may up-regulate tumor suppressor genes (Berger et al., 2011). Thus, also differentiation inducing liposomal encapsulated small oligonucleotide encoding small activating RNAs (MTL-CEBPA) are pro-anakoinotic drugs (Reebye et al., 2018; Sarker et al., 2018). Table V exemplarily summarizes first clinical results on MTL-CEBPA or ivosidenib, an IDH (Isocitrat-Dehydrogenase) inhibitor as differentiation inducing drugs.

In addition, metabolic modulators have been introduced, like metformin (Attia et al., 2008; Chae et al., 2016; Lecarpentier et al., 2017), which may also act transcriptionally (Coyle et al., 2016) and statins. IMiDs, COX-2 inhibitors and arsenic trioxide represent protein-binding pro-anakoinotic drugs.

Anakoinosis drugs include epigenetically acting agents, e.g., azacitidine, decitabine, valproic acid etc. acing broadly on chromatin, but also, (here, only mentioned) small molecules targeting specific epigenetic mechanisms, e.g., by inhibiting BET bromodomain transcriptional regulators; EZH2 (Enhancer of zeste homolog 2); DOT1L (DOT1-like, histone H3 methyl-transferase); IDH (Dawson et al., 2011; McCabe et al., 2012; Kim et al., 2015; Dang et al., 2016; Tögel et al., 2016; DiNardo et al., 2018).

The pro-anakoinotic activity of metronomic low-dose chemotherapies with their pleiotropic angiostatic, immunomodulatory, anti-inflammatory and drug specific effects, may be particularly exploited in combination with further master modulators (Hart et al., 2015). Clarithromycin, metronomically administered, shows similar activity profiles (Hart et al., 2015; van Nuffel et al., 2015; Romiti et al., 2017). Some pro-anakoinotic therapeutics on protein-binding level are arsenic trioxide, IMiDs, and COX-2 inhibitors are approved. Still in pre-clinical evaluation is a novel technology aiming at the targeted shut off transcriptional modulators with small molecules (Bradner et al., 2017; Tables 1, 2).

**Table 2:** The tool of anakoinosis inducing therapies may be separated as novel treatment pillar.

**Treatment paradigms/Diagnostics/Therapeutics**						
	**Three treatment pillars for systemic tumor therapy**					
Treatment characteristics	Classic targeted therapy: • Shutting off • Pathways • Cells	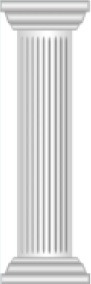	• Reactivating immune system • Classic targeted immunotherapy	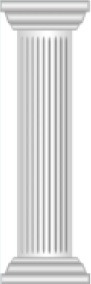	Anakoinosis: • Communicative reprogramming of tumor tissue and host	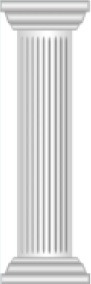
						
**Paradigms**	**Pathology**		**Pathology:**		**Pathophysiology:**	
	• Histology • Genome-centric: Uni-directional targeting		• Immune escape (multifactorial process)		• Multi-dimensional communication • Induction and perfection of evolutionary processes	
**Diagnostics**	**Situative snapshots**		**Immunologically accessible targets**		**Transcriptional dys-regulation**	
	• (Immuno-) histology • (Molecular-) genetics				• Homeostatic pathways • Tumor suppressor genes	
**Therapeutics**	**Targeted therapies:**		**Cellular therapies, antibodies etc.:**		***Master modulators***	
	• Blockade of pathways • Shutting off cells		• Modulation of immune response • Shutting off cells		• Of tissue homeostasis (poor monoactivity, but concerted activity)	

Frequently “old drugs” are used within pro-anakoinotic schedules in quite new functions, what is called “drug repurposing” (Bertolini et al., 2015). For the main part, the present review compiles drugs with poor monoactivity, particularly, also with respect to the scheduled dose reductions of single drugs for long-term administration (Hart et al., 2015).

The metronomic scheduling of drugs is an important component of pro-anakoinotic therapy approaches (André et al., 2017). Although in some tumor diseases rapid responses may be achieved within a 3–4-week cycle, other responses occur delayed, showing that a continuous systems therapeutic approach is necessary (Hart et al., 2015).

Clinical results of the reviewed therapeutic concepts integrating anakoinosis-inducing drug combinations indicate that features of palliative tumor care may diversified in a therapeutically meaningful way and that pro-anakoinotic schedules even have the capacity for inducing (continuous) complete remission (Hart et al., 2015; Thomas et al., 2015; Mayer et al., 2017).

### Reviewed pro-anakoinotic schedules for explicating the novel treatment approach

For explicating the novel methodological approach, we summarized data on the clinical administration of master modulators, i.e., transcriptional modulators in monotherapy or in various combinations (Table 1), (1) combined with metronomic low-dose chemotherapy, (2) pulsed chemotherapy, (3) demethylating agents, (4) classic targeted therapies or (5) protein-binding pro-anakoinotic drugs (arsenic trioxide, IMiDs, COX-2 inhibitors) (Tables I–VIII). Agonists and antagonists of transcriptional modulators, metronomic low-dose chemotherapy, epigenetically active agents, protein- binding pro-anakoinotic drugs, but also classic targeted therapies inducing for example differentiation (Table V) are considered as master modulators exploiting the tumors design space.

Available study data are unsuitable for presentation in a Cochrane or PRISMA analysis due to the diversity of schedules and the respective low patient numbers treated in each trial. Likewise, it would go beyond the scope of this study to give a comprehensive review on single master modulators.

Just the diversity of response patterns following anakoinosis-inducing schedules, their successful administration independent of tumor histology, the possibility for classifying responses according to operated communication tools, or for elaborating mechanisms of action, may highlight the sum of reported pro-anakoinotic treatment approaches as unique therapeutic pillar (Tables 1, 2).

Concerning transcriptional modulators, the explication of the novel methodological approach is restricted to selected transcriptional modulators in mono- or combination therapy, i.e., glitazones, glucocorticoids, vitamin D, somatostatin analogs, melatonin, interferon-alpha, estrogen, luteinizing hormone-releasing hormone (LHRH) agonist, retinoic acid and bexarotene.

For clarity, we do not consider (nuclear) receptor antagonists as clinical data on these drugs well established their clinical benefit, for example in breast and prostate cancer. In addition, not included are studies using COX-2 inhibitors plus pulsed chemotherapy, metformin or statins, also for reasons of comprehensibility.

For a better assessment of the clinical results on combinations of master modulators, particularly transcriptional modulators, additional data from randomized clinical trials are given comparing metronomic chemotherapy ± targeted therapies or metronomic with pulsed chemotherapy in Table VIII.

### Monoactivity of anakoinosis inducing drugs

Twenty-four reviewed studies included one transcriptional modulator, as monotherapy for cancer treatment, either a glitazone, a hormone or cytokine. Table 1 indicates the administered master modulators.

Monoactivity of glitazones or estrogen in cancer patients is very modest, whereas strong activity is well established in single tumor histologies for dexamethasone, LHRH agonist, somatostatin, and bexarotene (Tables IA/B, II, IV–VII; Querfeld et al., 2006; Sharma et al., 2008; Sidéris et al., 2012). The administration of interferon-α is superseded for melanoma or multiple myeloma (Browman et al., 1995; Di Trolio et al., 2015).

Oncological praxis does still not integrate metronomic low-dose chemotherapy, as routine therapeutic concept: Even combination therapies with classic targeted approaches or pulsed chemotherapy often show poor results in randomized comparison (Table VIII). Even more so, there is a growing number of combinatory schedules (Kerbel and Shaked, 2017), which shall bring to the fore the metronomic idea, meanwhile advanced in years, on the background of novel pharmacokinetic data (Bocci and Kerbel, 2016; Ciccolini et al., 2017).

Like metronomic chemotherapy, clarithromycin has a multi-functional activity profile and is currently being used in anakoinosis inducing schedules (ClinicalTrials.gov Identifier: NCT02852083) (Table 1; van Nuffel et al., 2015).

Demethylating and deacetylating agents show monoactivity in the range of commonly approved dose levels (Nervi et al., 2015). However, in anakoinosis inducing schedules much lower doses are going to be established (ClinicalTrials.gov Identifier: NCT02942758) (Thomas et al., 2015).

Agonists of “adopted” orphan receptors commonly have poor monoactivity in interventional cancer trials (Smith et al., 2004; Di Masi et al., 2015), in contrast to hormones and cytokines (McDermott et al., 2005; Mitchell et al., 2005). Particularly, dexamethasone plays a decisive role in the induction treatment for acute lymphocytic leukemia or multiple myeloma (Mitchell et al., 2005).

Metabolically active drugs, such as metformin or PPARγ/α agonists, are considered as chemopreventive agents (Fröhlich and Wahl, 2015; Higurashi et al., 2016). Metformin may prolong survival in cancer patients following surgery, but only in distinct histologic tumor types, as retrospective studies are indicating (Coyle et al., 2016).

Among the protein-binding drugs, arsenic trioxide and immunomodulatory imide drugs (IMiDs) have known monoactivity in hematologic diseases (Quach et al., 2010; Iland and Seymour, 2013) but both drugs are commonly administered combined with transcriptional modulators, all-trans-retinoic acid and dexamethasone, respectively (Lo-Coco et al., 2013; Benboubker et al., 2014).

### Simultaneous administration of two or more transcriptional modulators plus/minus additional master modulators (without metronomic low-dose chemotherapy)

Synergistic activity of dual transcriptional modulation has been well established in pre-clinical studies, for example for pioglitazone and all-trans retinoic acid in tumor cell lines of different histology (Papi et al., 2009, 2010, 2012, 2013), but also for glitazones in combination with chemotherapy (Elrod and Sun, 2008). Clinical trial designs translated these pre-clinical results hesitantly. Predominantly drugs, coming from immunomodulatory approaches (Il-2, interferon-α) found their way into combinatorial use (McDermott et al., 2005). Somatostatin analogs are administered besides their original application field, e.g., neuroendocrine tumors, also in castration-resistant prostate cancer, here in combination with estrogen or dexamethasone and LHRH analogs (Sciarra et al., 2004; Koutsilieris et al., 2006). In castration-resistant prostate cancer, the combination of transcriptional modulators alone may induce durable response (Table V; Koutsilieris et al., 2006). Combinations of estrogen with gestagen failed to show activity in endometrial carcinoma (Chlebowski et al., 2016).

Interestingly, interferon-α is active in renal cell carcinoma, both in combination with retinoids or pioglitazone (Buer et al., 1995; Walter et al., 2012; Aviles et al., 2015). Topical application of interferon-α and retinoids is helpful in corneal epithelial neoplasia (Krilis et al., 2012).

Standard schedules for the treatment of multiple myeloma include dexamethasone and IMiDs, also for maintenance therapy (Roussel et al., 2014).

### Transcriptional modulators combined with metronomic low-dose chemotherapy

Adding transcriptional modulators to metronomic low-dose chemotherapy schedules even led to complete remissions in a series of phase II trials for histologically quite different refractory tumor entities (Hart et al., 2015). Schedules included metronomic low-dose chemotherapy, COX-2 inhibitor and transcriptional modulators. In single patients with Langerhans cell histiocytosis, renal clear cell carcinoma, in epithelioid, less differentiated angiosarcoma, these schedules induced continuous complete remission (Vogt et al., 2003; Coras et al., 2004; Reichle et al., 2005; Heudobler et al., 2016).

It was only after the addition of interferon-α to metronomic chemotherapy plus pioglitazone and COX-2 inhibitor that continuous complete remissions occurred in metastatic renal clear cell carcinoma (Walter et al., 2012; Hart et al., 2015). This example clinically shows the pro-anakoinotic synergy effects of transcriptional modulators. Induction of continuous complete remission with dual transcriptional modulation only, supports current experimental data, showing that PPARγ plays neither a tumor-suppressive nor an oncogenic role in advanced renal clear cell carcinoma, and that single-agent PPARγ agonists are unlikely to be effective for the treatment of this disease (Sanchez et al., 2018).

**Figure 1:**
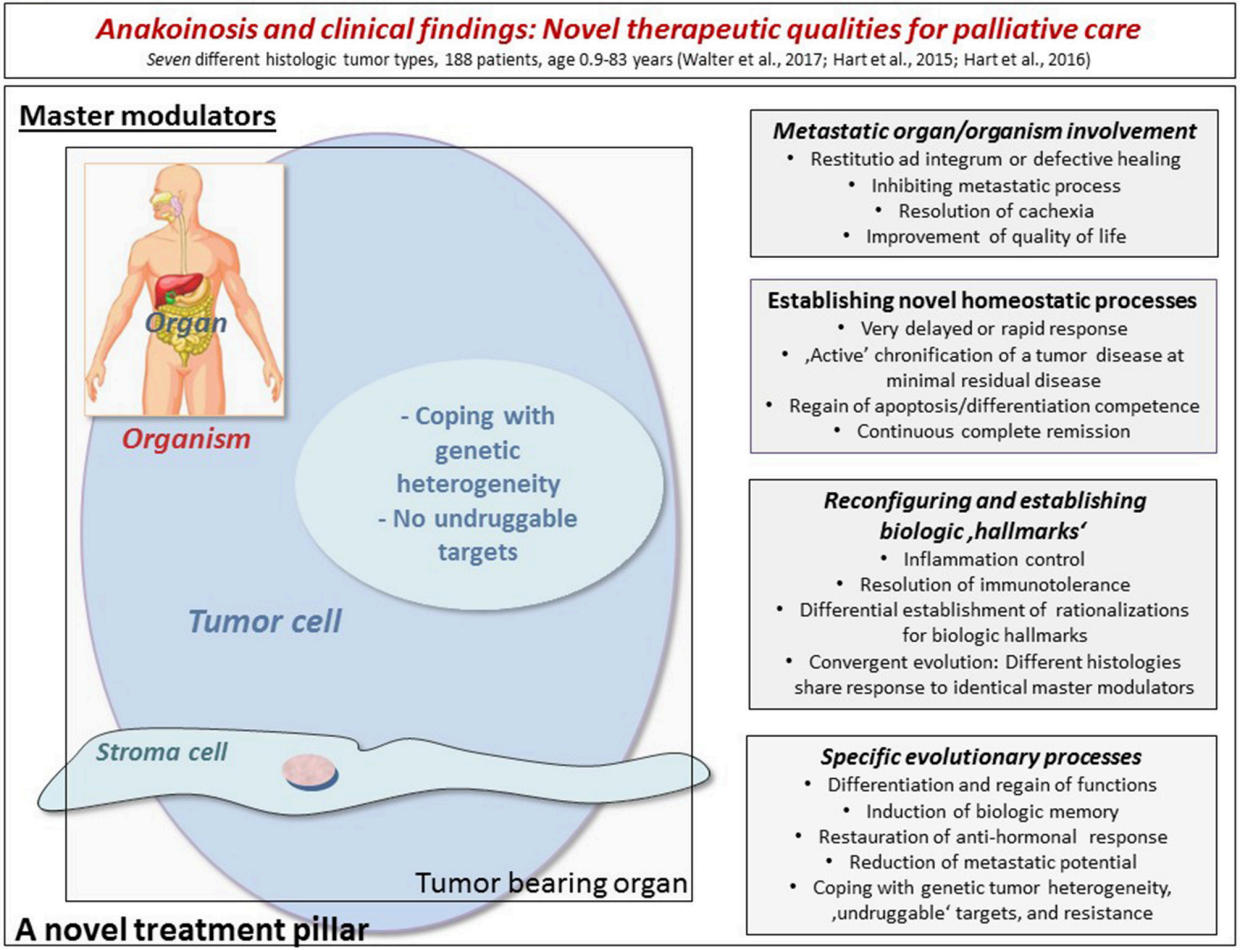
The figure indicates diversified outcomes in a summary of selected published studies on seven different histologic tumor entities (altogether 188 refractory and metastatic patients with age, ranging from 0.9 to 83 years). By modulating therapeutically accessible communication tools (left side), homeostasis mechanisms may be “normalized” in the tumor tissue, in the tumor-bearing organ and organism as indicated by a broad diversification of palliative tumor care or even continuous complete remission. The right side lists the multifaceted clinically observed phenomena during anakoinosis inducing therapies. The diversified clinical outcomes highlight clinical advantages compared to classic targeted therapies.

In a series of advanced and refractory hematologic diseases and solid tumors, combination therapies of pioglitazone with dexamethasone or interferon-α or all-trans-retinoic acid could be successfully used in addition to metronomic low-dose chemotherapy or azacitidine. Figure 1 indicates diversified outcomes in a summary of selected published studies on seven different histologic tumor entities (Walter B. et al., 2010; Walter et al., 2012; Hart et al., 2015, 2016; Thomas et al., 2015; Ugocsai et al., 2016).

Fenofibrate, retinoic acid, and vitamin D combined with metronomic chemotherapy induced in pediatric patients with refractory tumors of quite different histology complete remissions (15%) (Zapletalova et al., 2012).

Long-term responses at PSA levels <0.1 ng/ml occurred in rapidly progressive prostate cancer with PSA doubling times <3 months using combined transcriptional modulation, dexamethasone and pioglitazone in addition to metronomic low-dose chemotherapy. Pioglitazone may activate PPARγ, which is suggested to be a tumor suppressor in prostate cancer (Olokpa et al., 2017). The addition of imatinib in this schedule did not add any benefit. Interestingly, after discontinuing the study therapy due to non-tumor related surgery, patients remained at the status of minimal residual disease for up to 1.5 years. Such clinical observations are indicating anakoinosis as basis for the development of “biologic memory” in the anakoinotically modulated tumor tissue (Hart et al., 2015; Vogelhuber et al., 2015).

Besides nuclear receptor antagonists, LHRH agonists are standard therapies in breast and prostate cancer, mostly concomitantly integrated in therapy schedules (Graham and Schweizer, 2016; Nourmoussavi et al., 2017; Table VIII).

The worldwide alarming incidence of advanced liver cell carcinoma represents a great challenge for tumor therapy. An anakoinosis inducing therapy approach, including pioglitazone, COX-2 inhibitor and metronomic low-dose chemotherapy, has shown a comparably favorable influence on overall survival in advanced liver cell carcinoma, in comparison to more sophisticated systems therapies, including sorafenib, lenvatinib or anti-PD-1 antibodies (Walter et al., 2017). At that, an anakoinosis inducing therapy is cost-effective, and shows a lower toxicity rate. Therefore, a randomized comparison or a combination with targeted therapies seems to be a logical next step.

### Demethylation plus all-trans retinoic acid and pioglitazone or deacetylation plus bexarotene

The combination of azacitidine plus all-trans retinoic acid and pioglitazone may induce *ex vivo* granulocytic differentiation in more of 50% of blasts from acute myelocytic leukemia (Thomas et al., 2015). Moreover, these granulocytes regain phagocytic activity, when exposed to *E. coli* (Klobuch et al., 2018). Clinically, it is possible to induce continuous complete remission in acute myelocytic leukemia with the triple combination, while using only about 50% of the recommended dose of azacitidine.

Bexarotene, fenofibrate plus vorinostat may induce complete remission in tumor-stage mycosis fungoides (Steinhoff et al., 2008).

### Classic targeted therapies combined with anakoinosis inducing drugs

Among the combination of pro-anakoinotic substances combined with classic targeted therapies are several approved therapy schedules, interferon-α plus bevacuzimab (Escudier et al., 2007), proteasome inhibitors combined with dexamethasone, sandostatin plus mTor inhibitor, LHRH agonist plus tamoxifen (Tables I–VIII, brown). Classic targeted therapies may be successfully combined with anakoinosis inducing drugs, e.g., mTor-inhibitors in melanoma, or in 5th-line in refractory Hodgkin lymphoma, or imatinib in chronic myelocytic leukemia (CML), not achieving molecular complete remission, or bexarotene combined with denileukin difitox in cutaneous T-cell lymphomas (Foss et al., 2005; Hart et al., 2015; Prost et al., 2015; Ugocsai et al., 2016).

Dual metronomic chemotherapy combined with bevacizumab was efficacious in breast cancer: Higher baseline circulating endothelial cells correlate with significantly improved overall response and progression-free survival (Dellapasqua et al., 2008).

Although, representing a specifically targeted approach, CAR-T-cells act anakoinotic by infiltration, proliferation and cytokine storm in the tumor tissue (Chmielewski et al., 2014). Demethylating agents can efficiently modulate the immunophenotype of melanoma cells (Fratta et al., 2013).

### Randomized trials

Rosiglitazone, given as monotherapy, did not delay PSA progression in a placebo-controlled trial for prostate cancer (Smith et al., 2004).

In a randomized trial in T-cell lymphomas, dual transcriptional modulation with interferon-α plus retinoid is as potent as a chemotherapy containing regimen combined with interferon-α, but less toxic (Aviles et al., 2015). An analogous combination, interferon-α, pioglitazone plus metronomic low-dose chemotherapy, also shows synergistic effects in renal cell carcinoma in a historic comparison (Reichle et al., 2007a; Walter et al., 2012). Interferon-α has activity as maintenance therapy in lymphomas (Herold et al., 2015). The combination of interferon-α with lanreotide did not show any synergistic effects in neuroendocrine tumors (Faiss et al., 2003). Monoactivity of interferon-α is commonly weak among quite different tumor types (Eggermont et al., 2008). Therefore, the drug did not find its way in routine use or novel drugs are meanwhile more active, for example in melanoma (Agha and Tarhini, 2017).

Interestingly, the anakoinosis inducing combination of LHRH agonist, somatostatin analog plus dexamethasone is as efficacious as pulsed chemotherapy including estramustine and etoposide for treatment of castration-resistant prostate cancer (Dimopoulos et al., 2004).

A randomization in metastatic melanoma highlighted the addition of pioglitazone and COX-2 inhibitor to metronomic chemotherapy: The triple combination resulted in a significantly improved progression-free survival (Reichle et al., 2007b).

Although gastric cancer is commonly expressing PPARγ, the addition of pioglitazone and COX-2 inhibitor to metronomic chemotherapy did not improve outcome in a randomized comparison (Reichle and Hildebrandt, 2009).

Low-dose metronomic chemotherapy was not superior compared to pulsed chemotherapy in glioblastoma patients (Clarke et al., 2009). The addition of pioglitazone and COX-2 inhibitor to metronomic chemotherapy led to disease stabilization in heavily pre-treated patients with glioblastoma or astrocytoma (Hau et al., 2007).

In T-cell lymphoma, interferon-α plus retinoid may substitute low-dose MTX plus interferon-α (Aviles et al., 2015).

The combination arsenic trioxide and all-trans retinoic acid outcompeted ATRA plus chemotherapy for standard risk promyelocytic leukemia (APL) (Efficace et al., 2014).

### Anakoinosis inducing therapy concomitantly or sequentially to pulsed chemotherapy

Administered as adjuvant therapy, metronomic chemotherapy failed to show superiority, or patients only modestly benefit despite the addition of bevacizumab in osteosarcoma or colorectal cancer, respectively (Simkens et al., 2015; Senerchia et al., 2017; Table VIII).

Probably, raising vitamin D levels in serum from low/sub-normal to normal/high may contribute to a prolonged survival following chemo-immunotherapy in lymphomas (Bittenbring et al., 2014) whereas no advantage could be observed by the addition of vitamin D to docetaxel in prostate cancer (Attia et al., 2008).

The possible importance of normal or high vitamin D levels in serum on overall survival does not seem to be restricted to lymphoma patients treated with chemo-immune therapy, but there are also hints that patients following allogeneic blood stem cell transplantation may benefit from vitamin D substitution by a lower incidence of chronic graft-vs.-host reactions (Caballero-Velázquez et al., 2016).

Treatment of metastatic NSCLC with pulsed chemotherapy plus anakoinotically acting approaches including transcriptional modulators was less successful (Mantovani et al., 2000; Norsa and Martino, 2007). From available data one cannot conclude, whether the combination with pulsed chemotherapy may “destroy” anakoinotic activity profiles or the anakoinosis inducing “cocktail” was not chosen adequately adapted to the respective tissues' systems pathophysiology. The addition of a COX-2 inhibitor to pulsed chemotherapy did not significantly improve progression-free survival in first-line therapy for non-small-cell lung cancer (Edelman et al., 2017).

In contrast, metronomic chemotherapy plus combined transcriptional modulation may even induce complete remission in refractory tumors. Dual transcriptional modulation seems to be more efficacious as indicated by the response rate, long-term tumor control and continuous complete remissions (Reichle et al., 2007b; Hart et al., 2015).

In lymphoma patients, differential chemotherapy schedules may be associated with significantly different overall survival rates, irrespective of the fact that patients received identical prednisolone doses in each treatment arm (Bastion et al., 1997).

Interferon-α does not enhance activity of 5-fluorouracil (5-FU) in colon cancer (Hill et al., 1995; Thirion et al., 2001). Interferon-α plus retinoid adds no benefit to pulsed cisplatin in cervical cancer (Basu et al., 2016).

### Some combinations of transcriptional modulators are “supportive”

LHRH agonists are used for preserving fertility during pulsed chemotherapy (Taylan and Oktay, 2017), like osteoprotective regimens, such as LHRH agonist plus vitamin D (Scharla et al., 1990).

## Specific methodological aspects of anakoinosis inducing therapies

### What is the appropriate dosage of pro-anakoinotic therapy?

The question for the appropriate dose of each drug in an anakoinosis inducing schedule can be answered only pragmatically, based on clinical results and scheduled dose reductions, but currently, not yet pharmacokinetically (Hart et al., 2015; Bocci and Kerbel, 2016; Ciccolini et al., 2017; Walter et al., 2017).

Striking clinical results of anakoinosis inducing schedules are at first glance surprising on the background that the single drugs, particularly glitazones, have poor or no monoactivity. This comes true also for metronomic low-dose chemotherapy, as scheduled dose reductions have been performed within trials up to a dose, which would correspond to less than a quarter of the respective 3 weekly-administered cumulative dose (Hart et al., 2015; Walter et al., 2017).

Concertedly, pro-anakoinotic drug combinations may induce remissions, even continuous complete remissions, and may result in diversified palliative care strategies, as shown by multifold beneficial palliative effects in refractory neoplasia, as summarized in Figure 1. This way, the broad spectrum of available drugs, such as low-dose metronomic chemotherapy and transcriptional modulators come out from the corner as therapies validated as modestly efficacious: Once combined, these pro-anakoinotic treatment modalities (Tables 1, 2) have the capability for controlling refractory tumor disease. Thus, the drug combinations obviously act concertedly as regulators of the tumor cell systems, thereby, re-establishing apoptosis and differentiation competence in refractory tumor disease. Importantly, master modulators do not necessarily compromise non-diseased, homeostatically balanced organ systems, but may even improve organ functions (Hart et al., 2015; Walter et al., 2017). Possible side effects, allow an early scheduled dose reduction for avoiding further toxicity, and importantly, without significant loss of efficacy (Walter et al., 2017). Therapeutic efficacy despite dose reduction, particularly in case of metronomic low-dose chemotherapy, and the fact that, glitazones show no monoactivity in all available trials, are indicating the pro-anakoinotic, regulatory activity profile of the schedules (Table 1). We included metronomic low-dose chemotherapy also in the category “master modulators”: No evidence could be found in any reviewed clinical trial that scheduled dose reduction would have any impact on outcome–as far as transcriptional modulators have been additionally administered in the study schedule (Hart et al., 2015; Walter et al., 2017). Striking examples are the COMBAT trial for children with refractory tumors (Combined Oral Metronomic Biodifferentiating Antiangiogenic Treatment), and prospectively evaluated data on scheduled dose reduction of capecitabine in hepatocellular carcinoma; the schedule combined capecitabine etoricoxib and pioglitazone (Zapletalova et al., 2012; Walter et al., 2017). Reasons for a still available biomodulatory activity at very low-doses of metronomic chemotherapy could be synergisms between transcriptional modulators and metronomic chemotherapy: Pioglitazone, for example, generally up-regulates PTEN (Teresi and Waite, 2008; Berger et al., 2011), thereby, sensitizes hepatocellular carcinoma cell lines to 5-fluorouracil (5-FU), which is the active metabolite of capecitabine (Cao et al., 2007).

For monitoring anakoinosis inducing therapies it is meanwhile realistic to establish appropriate serum analytics (Mayer et al., 2017; Muqaku et al., 2017) to appreciate the functional status of cell systems and their changes (Pitteri et al., 2011). This way, we can approach the questions, which is the lowest, still regulative active dose of a single drug, and which are the most prominent players promoting tumor growth in the tumor tissue and the tumor bearing organ?

### Transcriptional modulation in cancer

Dysregulated transcription programs are an invariable consequence of oncogenic events, and represent the backbone of cancer (Berger et al., 2011). Dysregulation of transcriptional networks are the reason, why cancer cells are highly dependent on regulators of gene expression (Bradner et al., 2017). Gene regulatory network features reveal key regulatory networks and epigenetic changes that underpin tumor disease (Jin et al., 2018). Clusters of enhancers facilitate precise control of gene expression across normal cellular hierarchies, and are potential targets as central hubs in tumor disease (Bahr et al., 2018). The use of master modulators for treatment of metastatic refractory tumor disease, and the observed multifaceted clinically meaningful outcomes support the suggestion of hubs for regulation of transcriptional webs (Reichle and Hildebrandt, 2009; Hart et al., 2015; Huang et al., 2018).

Dysregulated transcriptional programs provide pivotal opportunities for a series of novel therapeutic interventions in metastatic refractory cancer (Giovannelli et al., 2012; Hart et al., 2015; Winter et al., 2015). Despite of currently expanding data, it is still difficult to broadly implement the current knowledge about transcriptional addiction for patients' benefit by targeting oncogenic transcription factors (e.g., PMLRARA), such as in case of acute promyelocytic leukemia (APL), resulting in substantial clinical benefit (Cicconi et al., 2016).

The targeted blockade of tissue-specifying nuclear transcription factors in cancer is well-established (estrogen, progesterone, androgen receptor) (Giovannelli et al., 2012). However, our knowledge of tissue-specifying transcription factors remains limited, and drugs with pro-anakoinotic activity, such as agonists of nuclear transcription factors regularly have an activity profile far above the capacity of hermeneutic comprehension (Reichle and Vogt, 2008).

Therapeutics, disrupting oncogenic transcription factors by targeted protein degradation, are still clinically not approved, but successfully studied in pre-clinical settings (Winter et al., 2015). In pre-clinical trials, blocking of super-enhancers of transcriptional networks is possible (Mack et al., 2018).

Master modulators in pro-anakoinotic therapy schedules evolve tumor systems in a therapeutically meaningful way by promoting communicative reprogramming, anakoinosis, implicating changes of validity and denotation of cell elements, and therefore also of cellular identity (Klobuch et al., 2018). Vice versa, neoplasia endogenously evolves, based on anakoinotic mechanisms. For example, hepatocellular carcinoma develops on basis of liver cirrhosis, or aging processes in normal B-cells precede B-cell chronic lymphatic leukemia. In case of cachexia, the tumor even affects the whole organism via reprogramming platelets, for example in metastatic melanoma (Mayer et al., 2017; Muqaku et al., 2017).

Transcriptional modulation with agonistic drugs, particularly dual transcriptional modulators, as discussed in the current review, showed clinically meaningful efficacy in a series of refractory metastatic neoplasia by communicatively reprogramming transcriptional networks maintained by tumor and adjacent stroma cells: Anakoinosis inducing approaches may re-adapt or “normalize” homeostatic pathways (Hart et al., 2015) by modulating functionally defined subsystems. Consecutively, subsystems may take over novel validity and denotation for constituting diversified rationalizations of biologic hallmarks (Box 1; Reichle and Hildebrandt, 2009).

Therapeutically intended transcriptional networking may have decisive regulatory impact on tumor promotion, for instance, on the angiogenic switch or on tumor stem cell behavior (Trosko, 2006). Targeting functionally defined subsystems with modulators of transcription factors seems to become of increasing interest, as subsystems within tumors may be exclusively functionally defined in a systems context but simultaneously linked to alternating structural systems (Pahler et al., 2008; Reichle and Hildebrandt, 2009).

### Characteristics of master modulators of tumor tissues

Contrary to the genetic, molecular genetic and phenotypic heterogeneity of metastatic tumor cells (Allgayer, 2018; Gerner et al., 2018); tumor growth-promoting sub-systems supporting hallmarks of cancer promise a high grade of similarities in constituting hallmarks of cancer or respective tumor-specific patterns of hallmarks. The successful administration of similar pro-anakoinotic schedules in refractory Hodgkin lymphomas and malignant melanomas, in castration-resistant prostate cancer and multiple myeloma, or in refractory angiosarcomas and Langerhans cell histiocytosis (Hart et al., 2015) underpins that tumors draw on a distinct repertoire of rationalizations supporting biologic hallmarks for constituting tumor phenotype (Box 1; Gerner et al., 2018). Thus, similar repertoires of drug combinations with pro-anakoinotic activity profile might be available, which target and regulate corresponding tumor-associated communicative subsystems mirrored for example by inflammation-related biomarkers etc. (Reichle et al., 2007a).

How can we integrate the finding that different histologic tumor types may share tumor response to distinct combinations of master modulators (Hart et al., 2015).

Recurring oncogenic events, such as alterations of NF-κB, TGFβ, Ras, p53, Myc, E2F/Rb/CDKN2, are associated with multifold tumor phenotypes (Ahmadiyeh et al., 2010): This observation explains that the tissue of origin predominantly characterizes tumor phenotypes as indicated by histology. The tumor phenotype depicts the recessive and thus, therapeutically accessible communicative interactions occurring during tumor evolution according to the restrictions given by the tissue-specific “metabolism” of evolution.

The tissue-specific “metabolism” of evolution makes different cancer types sharing similar tissue disruptions, alterations in homeostatic pathways and dysregulation of transcription factors, as tumor evolution deeply interweaves ubiquitously available wound healing mechanisms, inflammation, immunity, angiogenesis and metabolic processes (Virchow, 1859; Dvorak, 2015).

So-called super-enhancers spatially and temporally coordinate transcriptional webs for maintaining cell identity. Eqipollent, the neighboring environment communicatively mediates cellular identities (Reichle, 2013). Accordingly, synchronized “super enhancers” are responsible for higher order spatial re-organization of chromatin clusters that finally define cell identity (Hnisz et al., 2013). Oncogenic events must draw on such super-enhancers for establishing tumor-associated conditions described by the observation that tumors behave like never healing wounds (Virchow, 1859; Dvorak, 2015). Transcriptional super-enhancers may also explain convergence of developmental and oncogenic signaling pathways and their unique therapeutic accessibility (Hnisz et al., 2015).

Clinically observable changes in cell identities as indicated for example by differentiation of blasts from acute myelocytic leukemias and regain of granulocytic functions, suggest therapeutically important modifications of super-enhancers by pro-anakoinotic, transcriptionally active drugs (Hnisz et al., 2013; Hart et al., 2015; Sim et al., 2017; Klobuch et al., 2018). Figure 1 summarizes that cellular identity compromising tumor biologic changes are as expected, highly diversified, with strong impact on outcome. Interestingly, pre-clinical activities of inhibitors of super-enhancers among different tumor types are as multifaceted as clinical results presented in Figure 1 (Sengupta and George, 2017).

“Biologic memory” or long-term response in castration-resistant prostate cancer exemplifies the possibility for “active” chronifying tumor disease. Induction of molecular complete remission via differentiation induction could be observed in acute myelocytic leukemia or delayed objective response in renal clear cell carcinoma with consecutive continuous complete remission. Interestingly, combinatorial use of transcriptional modulators seems to be even more efficacious concerning outcome of refractory metastatic tumor disease (Koutsilieris et al., 2006; Hart et al., 2015).

An anakoinosis inducing schedule in hepatocellular carcinomas clinically demonstrated that the discrimination of local or metastatic disease has no significant impact on overall survival (Walter et al., 2017). By using the appropriate pro-anakoinotic therapy approach the suggested genetic heterogeneity at metastatic sites plays a minor role for outcome. These results highlight the novel pathophysiologic concept as basis for pro-anakoinotic therapies (Reichle and Hildebrandt, 2009).

Glitazones are the most frequently used transcriptionally active master modulators for anakoinosis induction besides glucocorticoids (Heudobler et al., 2018b). For example, pioglitazone, a peroxisome-proliferator-activated receptor (PPAR) γ/α agonist, seems to be an important many-sided applicable master modulator for communicative processes in neoplasia. Despite the missing monoactivity of glitazones in cancer (Table 1), pioglitazone is highly efficacious in combination with metronomic low-dose chemotherapy, or epigenetically active drugs (azacitidine), plus/minus further transcriptional regulators (interferon-α, dexamethasone or all-trans-retinoic acid (ATRA) (Tables I–VIII): After 3 years, the median overall survival has not been reached in a study on castration-resistant prostate cancer (Vogelhuber et al., 2015). In refractory Langerhans cell histiocytosis (Reichle et al., 2005; Heudobler et al., 2016), and (hem-) angiosarcomas continuous complete remissions have been observed (Vogt et al., 2003). Also in refractory acute myelocytic leukemia (Thomas et al., 2015) molecular complete remissions occurred, indeed by differentiation of blasts in phagocytic active neutrophils (Figure 1; Klobuch et al., 2018), but also by rescuing healthy hematopoietic maturation while repressing leukemic growth (Boyd et al., 2017). The addition of pioglitazone to imatinib in chronic myelocytic leukemias, not responding with molecular complete remission (MCR) may induce in a high percentage of patients MCR (Rousselot et al., 2017). In hepatocellular carcinoma, clinical data give hints that pioglitazone combined with metronomic low-dose chemotherapy and COX-2 inhibitor may communicatively reprogram in a clinically meaningful way the cirrhotic liver, tumor microenvironment and carcinoma cells (Walter et al., 2017).

Master modulators might interfere with master transcription factors and mediators establishing super-enhancers at key cell identity genes (Whyte et al., 2013). For example, PPARγ binds to the promoter of Dlc1 gene, a super-enhancer to regulate its expression during both white and brown adipocyte differentiation (Sim et al., 2017).

Performing studies on PPARγ in tumor cell cultures only, without co-culturing heterologous stroma derived cells leads to the conclusion that PPARγ may also function as a tumor promoter (Yun et al., 2018). Co-culturing tumor cells, however, with tumor-associated fibroblasts and dual stimulation with nuclear transcription factor agonists consistently results in tumor response within quite different histologic tumor types (Papi et al., 2009, 2010, 2012, 2013). *In vivo*, PPARγ agonists promote cell cycle arrest, cell differentiation (Klobuch et al., 2018; Ryu et al., 2018), and apoptosis and reduce inflammation (Hart et al., 2015), angiogenesis, oxidative stress, cell proliferation, invasion (Reichle and Vogt, 2008), and cell migration (Vallée and Lecarpentier, 2018).

Many pre-clinical data indicate that pioglitazone up-regulates non-mutated tumor suppressor genes and consecutively modulates homeostatic pathways in tumor tissues (Mulholland et al., 2005; Teresi and Waite, 2008; Berger et al., 2011; Rosner et al., 2017; Walter et al., 2017; Vallée et al., 2018). Mutations in the PPARγ gene are rare in neoplasia. Follicular thyroid cancer harbors a fusion gene (Eberhardt et al., 2010): A trial with pioglitazone is ongoing (ClinicalTrials.gov Identifier: NCT01655719).

Follow-up studies of the ProActive trial do not support any more the possibility that pioglitazone may favor the development of urothelial carcinoma in patients with diabetes mellitus type II (Erdmann et al., 2016).

### Pro-anakoinotic therapy schedules: indications and diagnostics

Transcriptional modulators are master modulators of tissue communication and are important pro-anakoinotic drugs, with obvious combinatorial activity among themselves, combined activity with metronomic low-dose chemotherapy, demethylating agents, pulsed chemotherapy or classic targeted therapy. It remains striking that the combinatorial use of the defined anakoinotic active drugs (Table 1) may induce (continuous) complete remission in refractory tumor disease.

All these observations prompt the assumption that irrespectively of the therapeutic technique for achieving apoptosis competence, either classically targeted, or with a cytotoxic approach, or by communicatively reprogramming a tumor disease, anakoinotic mechanisms are essentially necessary for finally initiating mechanisms, paving the way for continuous complete remission.

Assuming differential sequential biologic steps, necessary for achieving long-term tumor control in case of metastatic tumor disease (Beyar-Katz et al., 2016; Was et al., 2018), induction of anakoinosis opens the window for an active guidance and specified therapeutic support for re-establishing apoptosis or differentiation competence. Anakoinosis focuses on therapeutically guiding dynamic communication processes linked to tumor evolution or tumor response (Beyar-Katz et al., 2016; Was et al., 2018). More static evaluations of tumor characteristics, such as histology, immunohistology, molecular genetics and genetics serve as starting point for classic targeted therapies. Classic pathology-associated tumor evaluation includes parameters, which are mostly heterogeneous at metastatic sites (Figure 1).

In contrast, similarities of cellular immune response at primary site and metastatic sites of renal cell carcinoma or colorectal cancer (Remark et al., 2013) underline that tumor heterogeneity may be principally therapeutically overcome by pro-anakoinotic therapy approaches. Thus, evaluation of the respective “evolution-adjusted” tumor pathophysiology, such as homeostatic mechanisms, down-regulated tumor suppressor genes, transcriptional dysregulation etc., may in future diagnostically guide the selection of pro-anakoinotic schedules (Figure 1; Reichle, 2013).

Besides their function as rescue therapies for refractory neoplasia, anakoinosis inducing therapy schedules might supplement or substitute known consolidation therapies, such as continuous long-term maintenance therapy (Rousselot et al., 2017), adjuvant chemotherapies, or high-dose chemotherapy combined with autologous stem cell transplantation: Preliminarily results from clinical trials already indicate that anakoinosis inducing schedules may decisively influence long-term outcome (Tables I–VIII). For example, in chronic myelocytic leukemia without molecular remission (Rousselot et al., 2017) the addition of pioglitazone to imatinib may induce continuous complete remission: Even a discontinuation of imatinib and pioglitazone is possible. In patients with lymphoma serum vitamin D levels (Kelly et al., 2015; Prost et al., 2015) are predictive for overall survival. Lenalidomide is well established as maintenance therapy in multiple myeloma, all-trans retinoic acid in promyelocytic leukemia (Cicconi et al., 2016; McCarthy et al., 2017). Unfortunately, many chosen anakoinotic acting maintenance therapies showed modest improvement of progression-free survival, failed to demonstrate any important clinical effects or not used any more due to the availability of novel targeted therapies (Browman et al., 1995; Di Trolio et al., 2015; Simkens et al., 2015; Rochlitz et al., 2016).

Pro-anakoinotic therapy schedules might be the ideal adjuvants for classic targeted therapies efficaciously controlling, even eradicating disseminated neoplasia (Hart et al., 2015; Prost et al., 2015; Neelapu et al., 2017).

Generally, anakoinosis inducing therapies are characterized by a favorable toxicity profile (Dimopoulos et al., 2004; Zapletalova et al., 2012; Hart et al., 2015; Cicconi et al., 2016; Rousselot et al., 2017; Figure 1).

### Acknowledgment of a novel therapy concept

The review shows that even refractory metastatic tumor disease may respond to induction of anakoinosis: Moreover, independent of the histologic tumor type, anakoinosis inducing therapies are available for the successful treatment of refractory metastatic tumor stage (Figure 1).

Four communication-derived targets are accessible for anakoinosis-inducing therapy approaches: Figure 1 summarizes clinically observed events induced by anakoinosis in metastatic (refractory) neoplasia (Hart et al., 2015; Walter et al., 2017).

Clinical results from anakoinotic therapy approaches may topographically map four communication levels: Pro-anakoinotic combination therapies simultaneously modulate the single cell phenotype (Klobuch et al., 2018), but also tumor-stroma-cell, tumor-organ (Hart et al., 2016) and tumor-organism interactions (Muqaku et al., 2017), as particularly indicated in metastatic hepatocellular carcinoma or (uveal) melanoma (Hart et al., 2016; Walter et al., 2017). The clinical results depict also four communication tools, available for targeting: The holistic communicative system, i.e., specific tumor-stroma-organ-organism interactions, modularity, rationalizations, i.e., the physical constitution of biologic hallmarks‘, and the specific metabolism of evolution, given by a distinct cellular context at an organ site (Box 1).

Known prognostic parameters, such as therapy refractoriness in neoplasia, the specific mutations in Philadelphia chromosome in chronic myelocytic leukemia (Rousselot et al., 2017), FLT3 positivity in acute promyelocytic leukemia treated with all-trans retinoic acid plus arsenic trioxide (Cicconi et al., 2016), may ultimately lose their exclusive unfavorable prognostic significance by induction of anoikis, programmed cell death (Hart et al., 2015). Particularly, metronomic low-dose chemotherapy plus pioglitazone and COX-2 inhibitor separated PPARγ expression as late-stage prognostic parameter (Meyer et al., 2009).

The evidences are so strong as to indicate that anakoinosis represents a substantive therapy principle besides novel targeted therapies. Routine pathophysiological studies including homeostatic pathways, down-regulated tumor suppressor genes etc. are now in the center of diagnostic interest for guiding pro-anakoinotic therapy approaches. Here we still find a big diagnostic gap (Figure 1).

## Perspectives

Do tumor systems' complexity and the myriad of reductionist therapeutic approaches targeting separately tumor or stroma cells precede the simplicity of anakoinosis inducing treatment strategies (Reichle and Hildebrandt, 2009)?

Anakoinosis may induce a broad spectrum of palliative effects, moreover, continuous complete remission in refractory neoplasia (Hart et al., 2015). Indeed, anakoinosis inducing therapies may circumvent the problem of “undruggable targets” and cope with the general therapeutic problem of (molecular-) genetic tumor heterogeneity (Walter et al., 2017). In addition, pro-anakoinotic schedules may directly target tumor stem cells (Katoh and Katoh, 2007; Prost et al., 2015; Zhang et al., 2016; André et al., 2017).

The basic concept looms that fixed combinations of pro-anakoinotically acting drugs are available for treatment of quite different tumor histologies. Nevertheless, the respective therapy “cocktails” may be adopted according to specific, convergent organized systems rationalizations of cancer hallmarks, probably shared by different tumor histologies (Box 1; Hart et al., 2015). Pro-anakoinotic therapies may be specified according to the tumors' ontology (e.g., liver cirrhosis, myelodysplasia etc.), but also directed to the individual response patterns of the organism in response to the tumor disease, for example in case of cachexia (Hart et al., 2015; Muqaku et al., 2017). These multi-level communication tools are becoming accessible now by anakoinosis inducing therapy approaches, and spotlight again the impact of multi-dimensional communication levels on tumor promotion. We are just in the beginnings for appropriate diagnostic and therapeutic steps in this direction.

Anakoinosis inducing therapies successfully integrate many supplementary classic targeted approaches, as shown by initial results with mTor inhibitors, imatinib and bevacizumab, respectively (Table 1).

Anakoinotic processes may principally cope with fundamental obstacles of classic targeted therapies, with tumor heterogeneity and poor risk parameters, with context-dependent validity and denotation of tumor-promoting aberrations and targets, with drug resistance or undruggable targets by targeting dynamic evolutionary processes, for example multifaceted biologic steps necessary for establishing “active” long-term tumor control or continuous complete remission due to drug and tumor specific host responses (Box 1; Hart et al., 2015). Pro-anakoinotic therapies may inhibit further metastatic progression in case of metastatic disease (Figure 1; Reichle and Vogt, 2008).

A series of multicenter randomized phase II trials with anakoinosis inducing therapies, initiated by the University Hospital Regensburg, currently enrolling, include refractory acute myelocytic leukemia, castration-refractory prostate cancer, metastatic melanoma and non-small cell lung cancer. Large European trials on promyelocytic leukemia or chronic myelocytic leukemia are on the way.

Anakoinosis provides a novel therapy strategy for controlling even therapy-resistant metastatic tumor disease. The novel therapy principle draws on “old” drugs, and promotes drug repurposing in a rational way, oriented at recessive tumor associated events (Hart et al., 2015; Boyd et al., 2017; Walter et al., 2017).

Tissue engineering procedures may also implement anakoinosis for integrating multiple cell systems.

By introducing pro-anakoinotic schedules, innovation must not be adapted to local budget constraints to meet an area's need, e.g., in low-budget countries (Bouche, 2017), but may be universally applicable as novel therapeutic principle.

## Author contributions

DH, AR, LG, and CG conceived the meta-analysis. DH, LG, CG, and AR wrote the manuscript. LG and CG supported through interpretation of data for the work. All the authors revised the manuscript critically, approved the final manuscript, and agreed to be accountable for all aspects of the manuscript.

### Conflict of interest statement

The authors declare that the research was conducted in the absence of any commercial or financial relationships that could be construed as a potential conflict of interest.
